# Impact of family support improvement behaviors on anti diabetic medication adherence and cognition in type 2 diabetic patients

**DOI:** 10.1186/s40200-014-0113-2

**Published:** 2014-11-25

**Authors:** Hamidreza Khosravizade Tabasi, Farah Madarshahian, Mohsen Khoshniat Nikoo, Mohsen Hassanabadi, Gholamhossein Mahmoudirad

**Affiliations:** Birjand University of Medical Sciences, Ghafary Ave., Birjand, 9717853577 Iran; Endocrinology and Metabolism Research Center, Endocrinology and Metabolism Clinical Sciences Institute, Tehran University of Medical Sciences, North Kargar Street, Tehran, 1411413137 Iran

**Keywords:** Diabetes mellitus type 2, Family support, Medication adherence, Cognition disorder

## Abstract

**Background:**

Glycaemic control is the main goal of treatment for type 2 diabetic patients. Hyperglycaemia may result in cognitive decline. More family support may increase medication adherence and decrease glycaemic level. The purpose of this study was to determine the impact of family support improvement behavior on anti diabetic medication adherence and cognition in type 2 diabetic patients.

**Method:**

The randomized control trial study was conducted on 91 patients from an outpatient diabetes clinic. They were randomly divided to intervention (n = 45) and control (n = 46) group. Data on the patients’ demographic information and their family gathered using a questionnaire, For two groups Morisky Medication Adherence Scale (MMAS), drug administration part of Diabetes Social Support Questionnaire – family version (DSSQ), Number Connection Test (NCT) were applied and hemoglobin A1C was measured two times in the onset of study and three months later for control group and before and after intervention for intervention group. The key family members of the intervention group were taught according to their educational needs in small groups.

**Result:**

In intervention group mean of NCT score was significantly decreased after intervention (P = 0.006) however in the control group there was no significant difference after three months. In intervention group a significant correlation was noted between DSSQ scores and MMAS scores after intervention(r =0.67, P < 0.001) but, there was no significant correlation in the control group.

**Conclusion:**

Family support instruction based on the educational needs of family members, may improve medication adherence through direct effect and cognitive status with indirect effect.

## Introduction

Diabetes mellitus is a growing worldwide epidemic concern with an estimated number of 330 million by the year 2030 [1]. The results of epidemiological studies in Iran showed that the incidence of diabetes is 5%-8% (approximately 4 million people) of population [2].

Glycaemic control is one of the main goals of treatment for diabetic patients. Poor glycaemic control is a significant challenge in management of type 2 diabetes, with 36% to 69% of patients [3-6] failing to reach glycaemic control targets. Among effective factors on glycaemic control such as medication adherence, diabetes treatment, family support [7], factors that affect teaching learning process such as cognitive function [8-10] must be considered.

Chronic hyperglycaemia appears to be independently associated with cognitive function in individuals with diabetes [11]. Cognitive dysfunction in diabetic patients is known to have an important role on patients’ adherence to diabetes treatment [12]. Glycaemic level can cause slight or permanent Cognitive dysfunction and also diabetes can cause metabolic and micro vascular change on the brain and increase the risk of stroke [13]. Early and intensive glycaemic control is necessary to prevent or minimize the development of microvascular and macrovascular complications in individuals with type 2 diabetes mellitus [14]. Medication adherence is associated with improved outcomes in diabetes [15]. Nonadherence has been a recognized, persistent problem over the past 3 decades despite numerous informational, educational and behavioral interventions to promote better patients’ compliance, particularly with medication [16].

Medication adherence is a complex behavior related to and dependent upon many intrinsic patient risk and extrinsic (contextual) factors [17]. Adherence to prescribed medication requires a complex management process scheduling, adjusting to schedule changes, planning for availability of medication, remembering past events and problem solving around missed/late does [18]. While health care worker plays a role in delivering diabetes care and education patients, approach alone has been woefully inadequate [19]. Evidence suggests that family member can contribute to or buffer the deleterious effects of stress on glycaemic control. Although family members can provide many kinds of social support (e.g., emotional, informational and appraisal support) and instrumental support (e.g., observable actions that make it possible or easier for an individual to perform healthy behaviors) has been most strongly associated with adherence to self care behaviors across chronic disease [20].

Members of supportive families are more likely to have healthier behaviors, higher medication adherence and lower levels of stress that could explain their superior outcomes. Thus involving family members may improve the diabetes management. [21]. Although the results of some studies show that there is a correlation between degree of family support and clinical outcome, little research has focused on the effectiveness of family partnership intervention care for patient with poorly controlled type 2 diabetes [1] and also its indirect effect on improving cognitive status through a direct effect on increasing medication adherence. Family members may have a positive or negative effect on the self care of diabetic patients; therefore the purpose of this study was to determine the impact of family support improvement behaviors on anti diabetic medication adherence and cognition in type 2 diabetic patients.

## Methods

### Study participants and design

In this randomized clinical trial, 91 patients with type 2 diabetes who attended the diabetes clinic of the diabetes research center affiliated to Birjand medical university and lived with their family in 2013 were recruited (Birjand is one of the eastern cities in Iran with approximately 290000 people). Key family members were defined as those over 18 years old, having a blood relative and to be living with patient and were identified by the patient.

The inclusion criteria of the patients in the study were the age of over 30 years old, a history of type 2 diabetes more than one year (diagnosed by an endocrinologist), at least three last hemoglobin A1c reading equal or above 7% in the previous 12 months, besides no intention to change their lifestyle in the next 3 months (diet, exercise, medication, travel,), taking oral hypoglycaemic agents, insulin or both, and also more than 5 years schooling.

Those with severe vision and hearing problems, stroke, any endocrine disorder could interfere with cognition and severe cardiovascular diseases that could interfere with Number Connection Test (NCT) performance or were known risk factors for cognitive impairment were excluded. The intervention and control groups were comprised of 45 and 46 patients with type 2 diabetes respectively, who had medium or low adherence score according to Morisky Medication Adherence Scale (MMAS) [22], and had at least one “never” answer in the Diabetes Social Support Questionnaire (DSSQ) - family version [23].

Patients who met the inclusion criteria were randomly allocated to an intervention groups (n = 45) and control group (n = 46).

Participants were matched for sex, age, education, diabetes duration through group matching.

### Measurement and study variable

The patients’ demographic information, the number of family members who lived with them, type of treatment and “diabetes duration” (defined as the time between the diagnosis of diabetes mellitus and when NCT was performed) were extracted by one of the researchers. Data on the patients’ demographic information and family member gathered using a questionnaire.

MMAS is the 8-items easy to administered scale for medication adherence, with 7 yes/no items. Each yes response receives one point, while a no response receives 0 points. There is also one item with 5-point Likert-type response scales ranking from never to always. The response of never receives 1 point, while the remaining responses receive 0 point. The MMAS has a score range of 0–8, with higher numbers representing better adherence. The MMAS can also be categorized into 3 levels of adherence including high (score = 8), medium (score range, 6–7) and low (score, 0–5) [24,25]. The validity and reliability of MMAS has been approved in previous studies [24,25].

DSSQ-family version measures the perceived family support for adolescents diabetes care in four areas of diabetes management: drug administration, blood glucose testing, meals, and exercise. In this study drug administration domain, was considered and the patients only completed 8 questions of drug administration. The scoring was done based on 1–5 points dedicated to responses of never to always (Score range 8–40). The never response of the patients to at least one question stood for non supportive family in this study. The validity and reliability of DSSQ- family has been approved in previous studies [23].

A Number Connection Test was applied to assess the cognitive status (The mental process of acquiring knowledge and comprehension) of participants. This standard test consists of 25 circles distributed over a sheet of paper. It has different forms according to numbers and similar according to hardship. For performing this test, first the task to be accomplished was explained to the patient using demonstration test sheet then the second test sheet was completed by the patient. Test time was recorded by a stopwatch in seconds (15-30 = normal, 31-50 = slight, 51-80 = moderate, 81-120 = severe and upper to 120 = disable) [26,27].

All above examinations were performed for both intervention and control group in the onset of study. For participants who were in the intervention group, Educational needs on non supportive family items and key family member were determined. Then, the key persons of family members in intervention group divided to small groups (2–20 persons) according to their educational needs and were taught.

The instruction about the importance of medication adherence and family support behavior was carried out about 45–60 minutes in 3 sessions by one of the researchers. In every session 30–45 minutes considered for teaching and 15 minutes was allocated for answering the questions and exchanging of views between family members. The control group did not receive any instruction.

After 3 months, MMAS, DSSQ, NCT and hemoglobin A1C tests were performed in two groups. Venus blood samples for testing HbA1c were obtained after fasting and were analyzed on the same day in one laboratory.

### Statistical analysis

Data were analyzed using the statistical package for social science (SPSS software version 16, inc,chicaogo, IL, USA) in which p ≤ 0.05 was considered as statistically significant.

After determining the normality of data with Kolmogrove-Smirono test, data was analyzed for descriptive statistics, i.e. frequency distribution, mean and standard deviation and inferential statistics as follow:

Independent T test was used to compare age, duration of diabetes, hemoglobin A1c, NCT results, MMAS score, DSSQ scores between two groups and also, mean change of hemoglobin A1c, NCT score, MMAS score, DSSQ scores before intervention and after between two groups.

Paired T test was used to compare hemoglobin A1C, NCT results, MMAS score, and DSSQ scores before and after intervention for intervention group and first and second assess for control group.

Chi square test was used to compare the literacy status and sex between two groups. Pearson correlation test was used for DSSQ scores, and MMAS, NCT scores, hemoglobin A1c, age diabetes duration in two groups.

Fisher exact test was used to compare married status between two groups.

### Ethical aspect

This research was approved by ethic committee of Birjand university of medical science (no:9205). Each participant provided written consent. Participant data were associated with numbers rather than participant names.

## Results

Ninety one diabetic men and women (age range 34–74, mean age: 53.53 ± 7.58) participated in this research. Table 1 shows the demographic characteristics of the patients.

**Table 1:** **The demographic characteristics of the participants**

	**Intervention group N = 45**	**Control group N = 46**	**x2/T**	**P**
**Variable**				
**Age (y)**	52.93 ± 7.62	54.13 ± 7.56	−0.75	0.45
**Sex (n%)**				
**Female**	23(51.1)	25(54.3)	0.09	0.75
**Male**	22(48.9)	21(45.7)		
Married (n%)*				
**Married**	43(95.6)	46(100)		.024
**Other**	2(4.4)	0		
**Diabetes durstion (y)**	9.71 ± 6.75	11.39 ± 5.4	−1.31	0.19
**Education (n%)**				
**High school**	37(82.2)	36(78.3)	0.22	0.63
**Higher**	8(16.8)	10(21.7)		

*Result exact of Fisher test.

The spouses were reported as the main supporting person in the control and intervention group with a relative frequency of 89.1% and 75.6%, respectively.

The most frequency distributions of medication adherence items of MMAS in the intervention group before and after intervention were as follows:

A 46.7% rate of cutting back or stopping medicine treatment, due to no recovery, without informing the doctor reduced to 2.2%. Also, an occasional forgetting to take medication had a significant reduction from 91.1% to 22.2%. Likewise, feeling hassled about sticking to treatment plan had a reduction of 4.5%.

On the other hand, the distributions of participants’ adherence to prescribed medication based on the items of MMAS in the control group in the first and second assessment were like bellow:

Cut back or stopped taking medicine without consulting with the doctor, because of no improvement after medication, decreased from 39.1% to 26.1%. The occasional missing to take medicine increased from 73.9% to 80.4%. Feeling hassled about sticking to treatment plan had also an increase of 2/2% (from 84.8% to 87%).

The most frequency distribution of family support behavior items of DSSQ with “never” answer in intervention group before and after intervention respectively were: family members give you your medicine (reduction of 88.9% to 11.1%). Family members help out when you give yourself medicine (reduced rate of 77.8% to 11.1%). Family member wake you up so you can take your morning medicine on time (reduction of 88.9% to 33.3%).

The most frequency distribution of family support behavior items of DSSQ with “never” answer in control group in the first and second assessment were respectively: family members give you your medicine (reduction of 95.7% to 80.4%). Family members help out when you give yourself medicine (reduced rate of 80.4% to 71.2%). Family member wake you up so you can take your morning medicine on time (increased rate of 89.1% to 97.8%).

Table 2 shows the comparison of mean MMAS, DSSQ-family, cognitive, and hemoglobin A1C before and after intervention in intervention group and in first assess and after three months assessment in control group.

**Table 2:** **Comparison of mean variables before and after intervention in intervention group and in first and second assessment in control group**

**Group**	**Intervention group mean ± SD**		**n**	**T**	**P**	**Control group mean ± SD**		**n**	**T**	**P**
	**Before**	**After**				**First assess**	**Second assess**			
**Variable**										
**MMAS** (score)	3.1 ± 1.5	6.5 ± 1.6	45	−10.76	<.001	3.8 ± 1.0	3.3 ± 1.5	46	1.91	.06
**DSSQ **(score)	13.9 ± 6.5	24.8 ± 7	45	−8.67	<.001	14.3 ± 4.5	11.6 ± 3.2	46	3.96	<.001
**HbA1c **(%)	8.9 ± 1.3	7.7 ± 1.1	45	8.34	<.001	7.8 ± .7	8.1 ± .8	46	−2.27	.02
**NCT** (second)	170.9 ± 72.7	152.9 ± 46.3	45	2.89	<.006	189.2 ± 72.1	201.1 ± 68.7	46	−1.75	.08

MMAS: Morisky Medication Adherence Scale.

DSSQ: Diabetes Social Support Questioner.

NCT: number connection test.

In control group there was no correlation between DSSQ scores and MMAS scores, age, NCT scores and diabetes duration in first assess and after three months assessment.

In intervention group a significant correlation was noted between DSSQ scores and MMAS scores after intervention (r =0.67, P < 0.001) but, there was no correlation between DSSQ scores and age, NCT scores and diabetes duration before and also after intervention.

Table 3 shows a comparison of mean changes of DSSQ, MMAS, NCT and hemoglobin A1C before and after intervention in two groups.

**Table 3:** **Comparison of mean changes of DSSQ, MMAS, NCT and hemoglobin A1C before and after intervention in two groups**

**Group**	**Intervention group mean ± SD**	**Control group mean ± SD**	**T**	**P**
Variable				
MMAS(score)	↑3.3 ± 2.0	↓-.54 ± 1.9	9.25	<.001
DSSQ(score)	↑10.9 ± 8.8	↓-2.6 ± 4.4	9.57	<.001
NCT(second)	↓-18.0 ± 41.8	↑11.8 ± 45.6	−3.25	.002
HbA1c(%)	↓-1.2 ± .9 6	↑.3 ± .91	7.63	<.001

MMAS: Morisky Medication Adherence Scale.

DSSQ: Diabetes Social Support Questioner.

NCT: number connection test.

↑: Increase in mean.

↓: Decrease in mean.

## Discussion

The purpose of this study was to determine the impact of family support improvement behaviors on anti diabetic medication adherence and cognition in type 2 diabetic patients.

Family and social support are critical in managing diabetes treatment [28-30]. There is a lack of research regarding what family members know and feel about type 2 diabetes [31,32] and the potential influence of family members on the individual with type 2 diabetes has not yet been fully explored [33,34]in particular, the indirect effects of family members on cognition through improving medication adherence has not been well identified.

In this study, there was a significant difference between the mean score of family support (DSSQ) before and after intervention (P < 0.001) which increased to10.9 ± 8.84 after intervention. There was also a significant difference between the mean score of family support (P < 0.001) in two time assessments in the control group, but the mean change decreased to 2.63 ± 4.49 in 3 months later assessment. Most theories of health behavior changes require for diabetes self-care performance including a social support component [35,36] in which family members are all considered as a significant source of social support in adult with diabetes [31,37]. Family members can have a positive and or negative impact on the health of people with diabetes, interfere with or facilitate self care activities [38]. Greater social support has been shown to be associated with improved health outcomes and healthier behavior [39].

In this study decreased mean of family support in the control group may derive from the changeable supportive behavior of family members who don’t know how to help patients.

The results of this study indicated that there was a significant positive correlation between MMAS scores and DSSQ scores in intervention group while in control group there was no correlation. The parallel finding of previous study has shown that social support has been found to be an influencing factor on medication adherence, moreover members of more supportive families might have healthier behaviors, higher medication adherence and low levels of stress that could explain their superior outcome. Thus, involving family members may improve the management of diabetes [21]. The result of Scheurer et al. study evaluating the association between social support and medication adherence showed that, of 12 studies, 8 studies identified the significant association of support and adherence and for emotional support, of 14 studies, 6 surveys identified a significant association between any emotional support and adherence [39].

The results of present study showed the mean change of hemoglobin A1c decreased%1.2 ± 0.96 in intervention group and increased to%0.3 ± 0.91 in control group (p < 0.001). This finding may be as a result of better adherence to medication in intervention group. Mounting evidences show that psychological family-based intervention for patients with poorly controlled type 2 diabetes led to an improvement in glycaemic control [40].

This study revealed that the mean change of NCT score decreased in intervention group and increased in control group. Hyperglycaemia and high hemoglobin A1C levels [11] can cause cerebrovascular diseases and cognitive impairment [41]. Lowton et al. found that non adherence is more related to patient forgetfulness rather than to specific concerns about medications or interaction with physicians [1]. The condition along with chronic hyperglycaemia, commonly found in the sufferers, may result in cognitive improvement mainly in executive functions, memory, attention and psychomotor activity and disability in long term [11,42]. This can negatively affect the patient adherence to diabetes treatment [12]. Recent evidence suggests that the inclusion of a family member in psychosocial intervention chronic illness may improve illness outcome [43]. Many patients do not follow their medication orders and family support may improve the medication adherence, this may be a non-liner effect of family support on cognitive improvement through medication adherence.

## Conclusion

The results revealed that family support helps to improve medication adherence and cognitive status in patients with type 2 diabetes. Cognitive decline due to the presence of a risk factor of type 2 diabetes may occur as a result of hyperglycaemia. Family support may enhance medication adherence which results in glycaemic control and cognitive improvement as an indirect effect of family support. The results can help health care provider while counseling family member of people with type 2 diabetes. They can inform the family members about their effective roles and also teach them some techniques for improving their supportive behaviors.

It is suggested to study a naturally occurring social construct such as family support in patients with higher medication adherence and lower cognitive disorders to better understanding these conditions and apply it in artificially induced situations.
